# Skin scarification with *Plasmodium falciparum* peptide vaccine using synthetic TLR agonists as adjuvants elicits malaria sporozoite neutralizing immunity

**DOI:** 10.1038/srep32575

**Published:** 2016-09-14

**Authors:** Robert A. Mitchell, Rita Altszuler, Ute Frevert, Elizabeth H. Nardin

**Affiliations:** 1 Department of Microbiology, Division of Parasitology, New York University School of Medicine, New York, NY, USA

## Abstract

Malaria eradication will require a combination of vector control, chemotherapy and an easily administered vaccine. Sterile immunity can be elicited in humans by immunization with sporozoites, the infective stage injected by bite of the mosquito vector, however, whole parasite vaccines present formidable logistical challenges for production, storage and administration. The “gold standard” for infectious disease eradiation, the Smallpox Eradication Programme, utilized mass immunization using the skin scarification (SS) route. SS may more closely mimic the natural route of malaria infection initiated by sporozoites injected by mosquito bite which elicits both neutralizing antibodies and protective cell mediated immunity. We investigated the potential of SS immunization using a malaria repeat peptide containing a protective B cell epitope of *Plasmodium falciparum*, the most lethal human species, and delivery vehicles containing TLR agonists as adjuvants. In a murine model, SS immunization with peptide in combination with TLR-7/8 and -9 agonists elicited high levels of systemic sporozoite neutralizing antibody, Th1- type CD4+ T cells and resistance to challenge by bites of infected mosquitoes. SS provides the potential to elicit humoral immunity to target Plasmodium at multiple stages of its complex life cycle.

Experiments in the early 60’s and 70’s demonstrated that attenuated sporozoites delivered into the skin by the bite of irradiated Plasmodium-infected mosquitoes could elicit sterile immunity in experimental animals and human volunteers^1^,^2^,^3^. More recently, *P. falciparum* sporozoite induced sterile immunity has been confirmed in larger groups of volunteers and as few as 45 infected bites were found to elicit protective immunity in volunteers covered by chemoprophylaxis^4^,^5^,^6^. Multiple immune mechanisms have been shown to mediate protection in the sporozoite immunized experimental models, with antibodies that can immobilize sporozoites in the skin and block invasion of hepatic cells providing a first line of defense^7^,^8^,^9^,^10^. Sporozoites that evade antibody-mediated immunity and invade the host liver cells can subsequently be directly targeted by murine T cells^11^ which can also indirectly inhibit the development of the intracellular parasites by production of cytokines, such as IFN-γ^12^,^13^,^14^,^15^. Recent studies in sporozoite immunized mice have shown that CD11c+DC in skin draining lymph nodes can induce protective T cells that target the hepatic stage parasites^16^,^17^. Therefore, the skin potentially functions as a critical site both for the induction and the effector phase of adaptive immunity against the Plasmodium parasite.

A major target antigen of humoral and cellular sporozoite-induced immunity is the circumsporozoite (CS) protein that covers the surface of the Plasmodium sporozoite^18^,^19^. Vaccines comprised of synthetic peptides representing well-defined T and B cell epitopes of the *P. falciparum* CS protein have been shown to be safe and immunogenic in human volunteers, but these subunit vaccines required more potent adjuvants than the standard alum adjuvant found in the majority of licensed human vaccines (reviewed in ref. 20). More recent studies in the murine model have shown that adjuvant formulations containing synthetic TLR agonists can mimic signals provided by pathogen associated molecular patterns (PAMPs)^21^ and enhance immunogenicity of CS peptide vaccines^20^. In previous murine studies, we demonstrated that the topical application of the synthetic TLR7 agonist imiquimod, contained in an FDA-approved Aldara crème used for treatment for various skin diseases, can enhance the immunogenicity and protective efficacy of a malaria CS peptide vaccine administered by subcutaneous injection^22^. In recent clinical trials of a flu vaccine, the immunogenicity and protective efficacy of intradermally injected vaccine was enhanced by topical application of imiquimod at the injection site^23^,^24^, suggesting that studies of skin delivered vaccines in the rodent model are informative for development of human vaccines.

However, large-scale administration of vaccines via standard subcutaneous or intradermal injections in resource poor settings remains a challenge due to increased costs associated with the need for trained medical personnel and sterile syringes. We therefore examined malaria vaccine delivery via skin scarification (SS) using a bifurcated stylet as used for delivery of viral vaccine during the Smallpox Eradication Programme. SS with smallpox viral vaccine has been shown in recent murine and human studies to elicit superior responses relative to subcutaneous or intramuscular immunization, including ability to expand skin resident memory T cells (Trm) that disperse from the site of immunization throughout the skin and to other epithelial sites^25^,^26^.

To investigate the potential efficacy of SS delivered malaria vaccines, we utilized a peptide from the *P. falciparum* CS repeat region which contains the primary target of sporozoite neutralizing antibodies^27^. For the adjuvant formulation, we focused on TLR localized in the endosome by using the TLR 7 agonist imiquimod. TLR 7/8 agonist resiquimod, TLR 3 agonist poly I:C, and TLR 9 agonist CpG ODN, with the potential to enhance immune responses by co-localization of antigen and TLR signal within the endosome^28^,^29^. The goal of these studies was to determine if SS delivery of a *P. falciparum* CS vaccine with TLR agonists could elicit neutralizing antibodies that block sporozoite invasion *in vitro* and *in vivo* and protect mice against challenge by bites of mosquitoes infected with transgenic rodent parasites expressing *P. falciparum* CS repeats.

## Results

### Skin scarification (SS) with malaria peptide in Aldara elicits Th1- type immune responses

**Figure 1:**
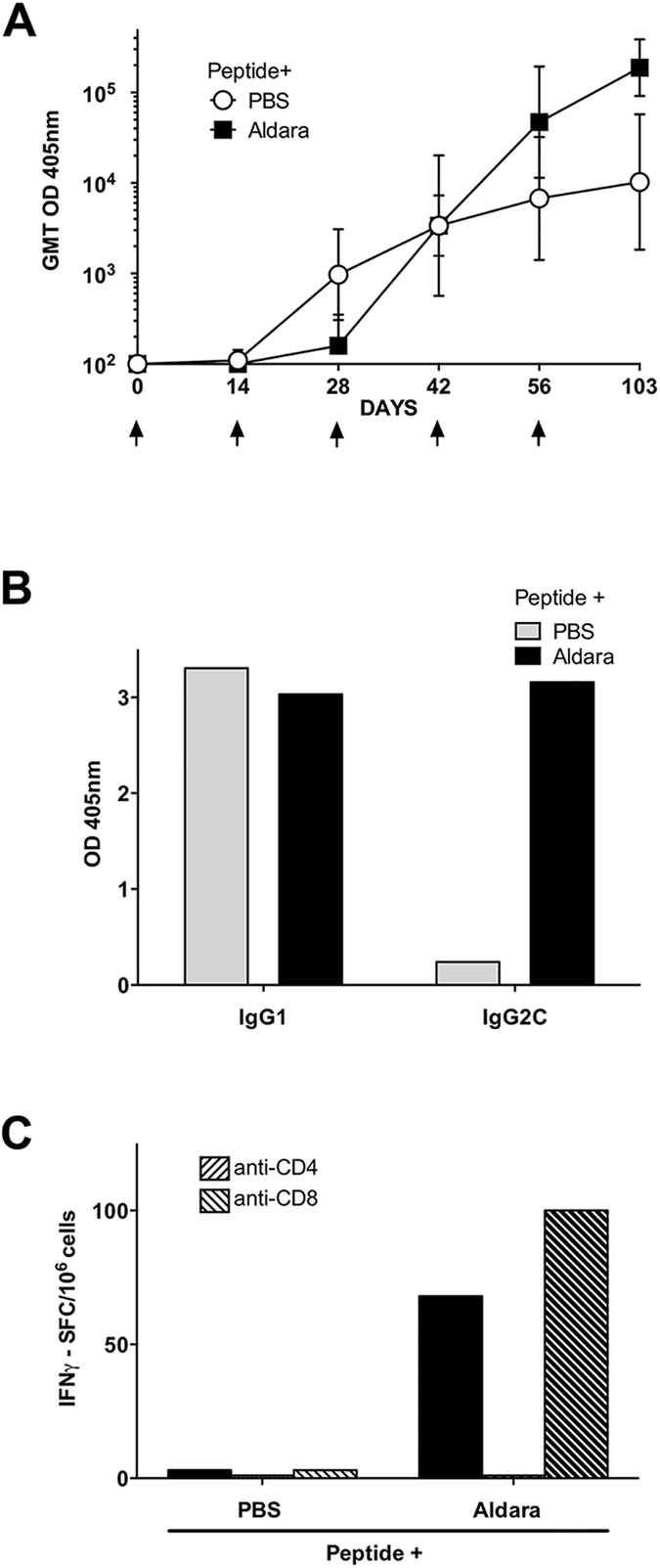
Skin scarification (SS) with malaria peptide in Aldara adjuvant elicits Th1-type antibody and cellular responses. (**A**) Kinetics of IgG antibody response measured in ELISA using serum of C57Bl/6 mice immunized at two week intervals by SS with a *P. falciparum* CS peptide in PBS or in Aldara containing imiquimod, a TLR 7 agonist. Individual serum of 5 mice/group obtained +14 days post each immunization (arrows) was tested by ELISA and results shown as geometric mean titer (GMT). (**B**) IgG subtypes were measured by ELISA using pooled immune serum (1:320 dilution) and MAB specific for murine IgG1 or IgG2c. (**C**) CS-specific T cells were measured in spleen cells obtained after the final SS immunization using IFNγ or IL-5 ELISPOT. Results are shown as IFNγ SFC/10^6^ spleen cells stimulated with repeat peptide in the absence (solid bars) or presence of MAB specific for CD4 or CD8 molecules (hatched bars). No malaria specific SFCs were found in Adjuvant only control group (data not shown).

To assess immunogenicity of malaria vaccine delivered by SS, C57Bl/6 mice were immunized by SS of scapular dorsal skin with *P. falciparum* CS repeat peptide without adjuvant (PBS) or mixed with Aldara containing the TLR 7 agonist imiquimod (Fig. 1A). SS with CS peptide without adjuvant elicited low anti-repeat IgG antibody titers (GMT 3,378) after three immunizations, which were not significantly increased (>4-fold) by additional SS boosters, reaching GMT 10,240 following the fifth dose. In contrast, SS with CS peptide in Aldara elicited a log higher peak IgG, with anti-repeat GMT of 188,203 following the fifth SS immunization. Moreover, in contrast to the Th2-type IgG1 antibody response elicited by SS with CS peptide in PBS, CS peptide in Aldara elicited a mixed Th1/Th2 type antibody response comprised of IgG2c as well as IgG1 subtypes (Fig. 1B).

Consistent with a shift to a Th1-type IgG2c antibody, spleen cells of mice immunized by SS with CS peptide in Aldara had IFNγ secreting CS-specific T cells (Fig. 1C), with little or no IL-5-producing cells (data not shown). The IFNγ producing cells were CD4+ T cells, as shown by reduction in number of IFNγ spot forming cells (SFC) in the presence of anti-CD4 MAB but not anti-CD8 MAB (hatched bars). Spleen cells of mice immunized by SS with CS peptide in PBS did not have detectable IFNγ SFC, consistent with skewed Th2-type IgG1 antibody in sera of these mice.

**Figure 2:**
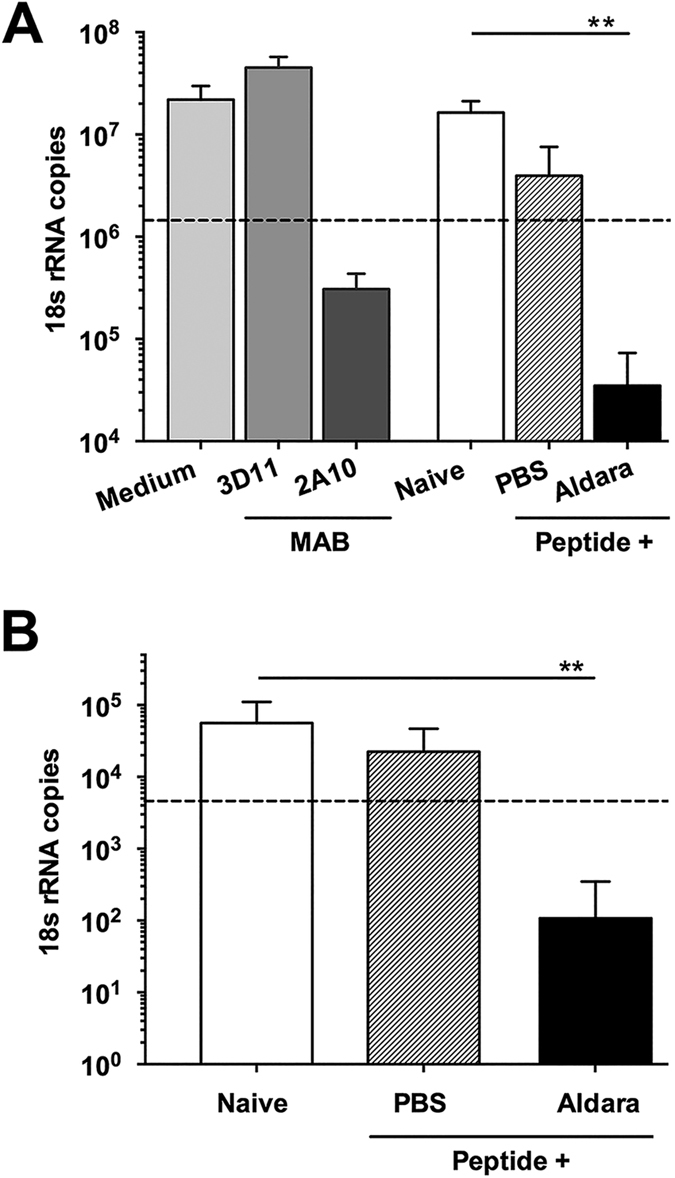
SS with *P. falciparum* CS peptide in Aldara elicits sporozoite neutralizing antibodies. (**A**) Antibodies were measured *in vitro* using TSNA and individual serum (1:5 dilution) obtained following five SS doses of CS peptide with or without Aldara. Results are shown as mean number of transgenic PfPb parasite 18S rRNA copies in HepG2 cell cultures at 48 h. post infection as measured by real-time qPCR. MAB 2A10 (25 μg/ml), specific for *P. falciparum* CS repeats, provided a positive control for > 90% inhibition of parasite burden (dotted line). Negative controls included PfPb sporozoites in medium, naïve serum or with MAB 3D11 specific for *P. berghei* CS repeats. (**B**) Following five SS immunizations, mice were challenged by exposure to the bites of 10–15 PfPb infected mosquitoes. Levels of parasite 18S rRNA were measured by qPCR in extracts of liver obtained 40 h. post challenge. Results shown as mean parasite 18S rRNA copy number in each group, with dotted line indicating 90% reduction of parasite rRNA copy numbers. P < 0.05 by Kruskal-Wallis test with Dunn’s multiple comparison between groups against naïve. Error bars are SD for all qPCR replcates.

Importantly, the anti-repeat antibodies elicited by SS with CS peptide in Aldara were functional both *in vitro* and *in vivo.* Incubation of transgenic *P. berghei* sporozoites that express *P. falciparum* CS repeats (PfPb) with serum from mice immunized by SS with CS peptide in Aldara, neutralized sporozoite infectivity for hepatoma cells, as shown by >90% reduction in parasite rRNA detected in hepatoma cell cultures at 48 h after infection (Fig. 2A). The neutralizing activity of the immune serum was comparable to inhibition obtained by pre-incubation of the PfPb sporozoites with 25 μg/ml MAB 2A10 specific for *P. falciparum* CS repeats. Neutralizing antibodies were specific for *P. falciparum* CS repeats as MAB 3D11, specific for *P. berghei* repeats, did not inhibit infectivity of PfPb sporozoites. Serum from mice immunized SS with peptide in PBS did not inhibit sporozoite invasion *in vitro*, giving parasite 18S rRNA copy numbers that were not significantly different from those obtained with naïve serum or PfPb sporozoites without serum (medium control).

The presence of sporozoite neutralizing antibodies in immune serum predicted resistance of the immunized mice to challenge by the bite of PfPb-infected mosquitoes (Fig. 2B). There was >90% reduction in parasite 18S rRNA in liver cell extracts of challenged mice immunized by SS with CS peptide in Aldara when compared to levels in naïve mice (p < 0.001). A reduction of >90% of parasite liver burden is considered biologically significant based on previous studies showing a delayed prepatent period or absence of infection using known numbers of injected sporozoites^1^,^30^. In contrast, following challenge of mice immunized by SS with CS peptide in PBS, parasite 18S rRNA copy numbers were not significantly different from naïve mice, consistent with the lack of detectable sporozoite neutralizing antibodies in the sera of these mice.

These initial experiments demonstrated that SS with CS peptide in Aldara could elicit neutralizing antibodies and reduction of parasite burden *in vivo* similar to that observed in our previous studies using topical Aldara applied to the site of subcutaneously injected peptide^22^. However, in both experimental regimens a total of five doses were required to elicit high levels of sporozoite neutralizing antibodies. We therefore explored whether immunogenicity of SS delivered vaccine could be enhanced by adding other TLR agonists to the TLR 7 agonist imiquimod contained in Aldara crème formulation.

The synthetic dsRNA polyriboinosinic:polyribocytidylic acid (poly I:C), which targets the endosomal TLR 3 that signals through the TRIF pathway, has been shown to be a potent adjuvant for a recombinant CS protein^31^,^32^. SS immunization with CS peptide in Aldara plus the TLR 3 agonist poly I:C increased the number of IFNγ secreting CD4+ T cells detectable in the spleen (Supplementary Fig. S1). However, SS with Aldara+poly I:C did not increase anti-repeat antibody titers compared to Aldara alone with GMT of 65,020 versus 163,840, respectively. SS with CS peptide and poly I:C only, elicited low Th2-type IgG1 anti-repeat antibody, similar to antibody elicited by CS peptide in PBS. Significant levels of sporozoite neutralizing antibody (>90% inhibition) were obtained only with serum from mice immunized SS with CS peptide in Aldara (Supplementary Fig. S1D).

### SS with malaria peptide in AddaVax elicits Th2- type immune responses

A limitation of the commercial Aldara formulation was the presence of numerous excipients including emulsifiers, permeation enhancers and preservatives^33^ that could potentially modify the function or interaction of the added TLR agonists. We therefore examined whether AddaVax, a squalene oil-in-water emulsion comparable to the MF59 adjuvant licensed for seasonal flu vaccine^34^ could be used for SS delivery of CS peptide and TLR agonists. In initial experiments, we compared immune responses elicited by SS with CS peptide in Aldara to SS with CS peptide in Addavax, with or without the addition of the TLR 7 agonist imiquimod or the TLR 3 agonist poly I:C. A maximum of four doses were tested as a more sensitive assessment of immune potentiation.

**Figure 3:**
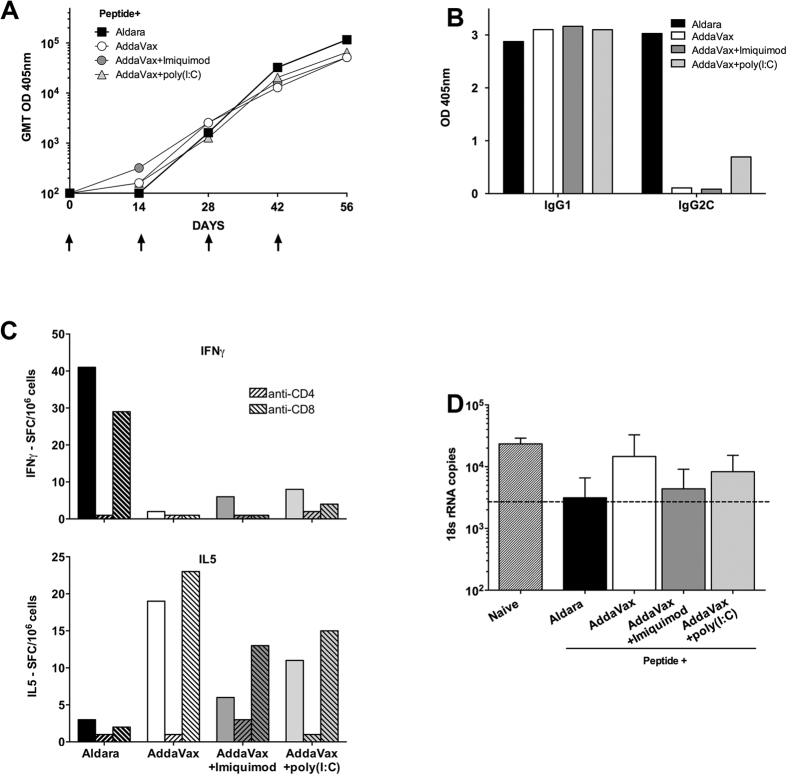
SS with CS peptide in AddaVax, with or without TLR 7 agonist imiquimod or TLR 3 agonist poly I:C, elicits primarily a Th2-type response and minimal protection. (**A**) Kinetics of IgG anti repeat antibody GMT measured by ELISA using individual serum (5 mice/group) obtained following four SS immunizations (arrows) with CS peptide in Aldara, containing the TLR7 agonist imiquimod, or in AddaVax with or without addition of TLR 7 agonist imiquimod or TLR 3 agonist poly I:C. (**B**) IgG subtypes of anti-repeat antibodies in pooled serum (1:320 dilution) obtained following four SS doses with peptide in Aldara or Addavax with or without imiquimod or poly I:C. (**C**) IFNγ ELISPOT (upper panel) and IL-5 ELISPOT (lower panel) of pooled spleen cells (3 mice/group) obtained following four SS immunizations. Results shown as SFC/10^6^ spleen cells in the absence (solid bars) or presence of MAB anti-CD4 or anti-CD8 (hatched bars). (**D**) Mice were challenged by exposure to the bites of PfPb infected mosquitoes following four SS immunizations. Results shown as mean 18 S rRNA copy number in liver extracts prepared 40 h post infection as measured by qPCR. Kruskal-Wallis test with Dunn’s multiple comparison between groups against naïve; no significant difference was detected between groups. Error bars are SD for all qPCR replicates.

The anti-repeat antibody responses elicited following SS immunizations with CS peptide in AddaVax were similar to Aldara, with peak GMT 51,606 and 115,852, respectively (Fig. 3A). The antibody titers were not enhanced following addition to AddaVax of either imiquimod (GMT 51,606) or poly I:C (GMT 65,020). In contrast to the mixed Th1 and Th2–type antibody response elicited by Aldara, SS with CS peptide in Addavax with or without imiquimod or poly I:C, elicited a predominantly Th2-type IgG1 antibody response (Fig. 3B). Consistent with the skewing of the IgG1 antibody response, the CD4+ T cells of mice immunized by SS with AddaVax with or without the TLR agonists secreted predominantly IL-5, with minimal IFNγ-secreting CD4+ T cells (Fig. 3C). As found in previous experiments, SS with CS peptide in Aldara elicited IFNγ-secreting CD4+ T cells, with minimal or no IL5-secreting T cells, consistent with the induction of Th1-type IgG2c antibody response.

Four doses of Aldara formulation gave suboptimal protection following challenge by exposure to the bites of PfPb infected mosquitoes (Fig. 3D). There was no enhancement of protective immunity in mice immunized by SS with CS peptide formulated in AddaVax alone, or following the addition of the TLR 7 agonist imiquimod or the TLR 3 agonist poly I:C to the oil adjuvant.

### SS with malaria peptide in AddaVax containing a combination of TLR 7/8 and TLR 9 agonists elicits enhanced sporozoite neutralizing antibodies

**Figure 4:**
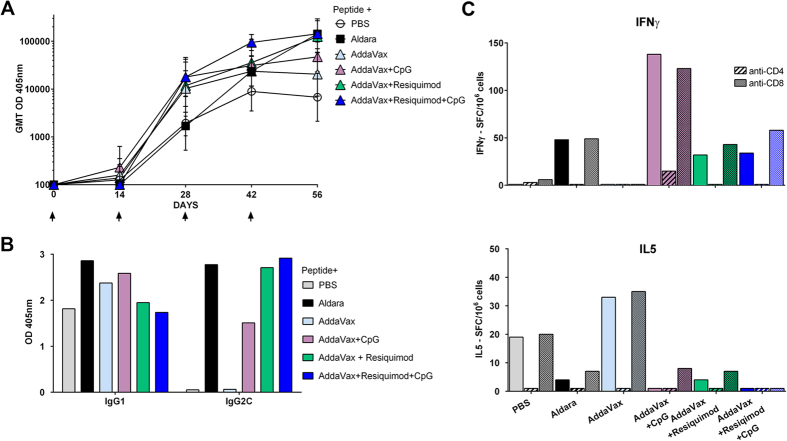
SS immunization with CS peptide in AddaVax containing TLR 7/8 agonist and/or TLR 9 agonists elicits Th1- type antibody and CD4+ T cells. (**A**) ELISA IgG anti-repeat GMT shown for serum collected+14 days post each SS immunization (arrows) with CS peptide in PBS, in Aldara or in AddaVax with or without the TLR 7/8 agonist resiquimod, the TLR 9 agonist CpG ODN or a combination of resiquimod and CpG. (**B**) IgG subtypes in pooled sera (1:5120 dilution) obtained following the fourth SS immunization with CS peptide in PBS, Aldara or Addavax with or without resiquimod, CpG or a combination of resiquimod and CpG. (**C**) CS-specific T cells in pooled spleens (5 mice/group) obtained post the fourth SS immunization as measured by IFNγ ELISPOT (upper panel) and IL5 ELISPOT (lower panel). No malaria-specific SFCs were found in spleen cells obtained from Adjuvant only control group (data not shown). The cellular source of cytokine was determined by inclusion of MAB anti-CD4 or anti-CD8 (hatched bars).

Recent studies in nonhuman primates with an HIV gp140 recombinant protein have shown that a combination of TLR 7/8 and 9 agonists formulated in a squalene oil adjuvant elicited enhanced levels of functional antibodies following intramuscular immunization^35^. We therefore examined whether SS immunization with CS peptide in AddaVax containing the TLR 7/8 agonist resiquimod and/or the TLR 9 agonist CpG could enhance immunogenicity and protective humoral immunity against sporozoites. Following four SS immunizations, AddaVax containing the combination of TLR agonists elicited anti-repeat antibody titers comparable to Aldara, with GMT 142,631 (Fig. 4A). In contrast to the skewed Th2-type antibody elicited by SS with AddaVax, SS with AddaVax containing either the TLR 7/8 agonist resiquimod and/or the TLR 9 agonist CpG shifted the response to a Th1-type IgG2c antibody (Fig. 4B; Supplementary Fig. S2). Consistent with the shift in antibody subtype, the addition of resiquimod and/or CpG to AddaVax elicited IFNγ- secreting CD4+ T spleen cells (Fig. 4C). SS with AddaVax without TLR agonists elicited predominantly Th2-type IL5-secreting CD4+ T cells, consistent with the skewed IgG1 antibody response found in sera of these mice.

**Figure 5:**
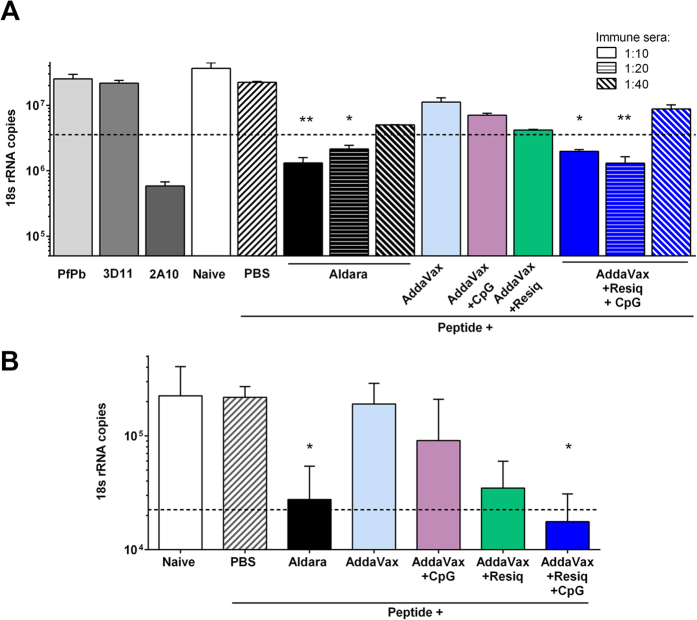
SS with CS peptide in AddaVax containing a combination of TLR 7/8 agonist and TLR 9 agonists elicits enhanced levels of sporozoite neutralizing antibodies and protection. (**A**) TSNA was carried out by incubating PfPb sporozoites with 1:10–1:40 dilutions of immune serum pools obtained from mice immunized SS with four doses of CS peptide in Aldara or in AddaVax with or without resiquimod and/or CpG. TSNAs were carried out three times with similar results and results shown for representative assay. (**B**) Mice were challenged by exposure to bites of PfPb infected mosquitoes and parasite levels were measured by real-time qPCR in liver extracts obtained 40 h post challenge. *P < 0.05; **P < 0.01 by Kruskal-Wallis test with Dunn’s multiple comparison between groups against naive. Error bars are SD for all qPCR replicates.

While AddaVax containing either resiquimod or CpG enhanced immune responses, SS with CS peptide in AddaVax containing a combination of TLR 7/8 agonist and TLR 9 agonists elicited higher levels of functional immunity (Fig. 5). Significant levels of sporozoite neutralizing antibodies were detected at 1:10 and 1:20 dilution of serum of mice immunized SS with CS peptide in AddaVax containing both TLR agonists (p < 0.05), comparable to inhibition in sera from mice immunized by SS with CS peptide in Aldara (p < 0.01) (Fig. 5A). In contrast, no sporozoite neutralizing antibodies were detected in 1:10 dilution of sera from mice immunized SS with CS peptide in AddaVax containing either TLR agonist alone. Increased sporozoite neutralizing antibodies elicited by SS with the combination of TLR as compared to single TLR was also detected in a second experiment when serum of individual mice were tested at 1:5 dilution (Supplementary Fig. S3).

The higher levels of sporozoite neutralizing antibodies in serum of mice immunized by SS with CS peptide in Addavax plus the combination of both TLR 7/8 and TLR 9 agonists (Fig. 5A) correlated with >90% reduction in parasite burdens in the livers of these immunized mice following challenge by exposure to the bites of PfPb infected mosquitoes (Fig. 5B) (r^2^ 0.834, p = 0.03). Following challenge, 60% (3/5) of the mice immunized by SS with CS peptide in Addavax plus the combination of TLR agonists had >90% reduction in parasite burden. Similarly, 60% (3/5) of mice immunized SS with CS peptide in Aldara had >90% reduction in parasite burden. In contrast, mice immunized by SS with CS peptide in AddaVax alone, or AddaVax plus a single TLR agonist, had 18S rRNA copy numbers that were not significantly different from controls following challenge.

In an effort to define the characteristics of sporozoite neutralizing antibodies in mice immunized by SS with CS peptide in AddaVax containing a combination of TLR agonists, as compared to AddaVax with a single or no TLR agonists, we examined anti-sporozoite antibody fine specificity using various serological assays (Table 1). The magnitude of peak anti-repeat GMT was similar in AddaVax formulations containing a single versus a combination of TLR agonists, with GMT not differing by >4-fold. While a combination of TLR agonists has been shown to broaden the fine specificity of the antibody response in other models^36^,^37^, no significant difference in the levels of antibody specific for major or minor repeats was observed in serum of mice immunized by SS with CS peptide in AddaVax containing single as compared to a combination of TLR agonists. In addition, the affinity of anti-repeat antibodies, as measured by chaotropic elution assays, did not differ in the serum of mice immunized by SS with CS peptide in AddaVax containing either a single or the combination of TLR agonists (Table 1, Supplementary Fig. S4). Similarly, antibodies that efficiently cross-link CS on the surface of viable sporozoites, as measured by the circumsporozoite precipitin (CSP) assay^38^, were of similar titer in mice receiving single as compared to the combination of TLR agonists (Table 1). In addition, a competition assay to measure ability of immune serum to compete with MAB 2A10 for binding to CS repeat peptide detected similar IC_50_ for AddaVax+ resiquimod immune serum (16.7 ug/ml) and AddaVax+ resiquimod+ CpG immune serum (14.4 ug/ml) (Supplementary Fig. S5) despite functional differences in the sporozoite neutralizing activity of these immune serum *in vitro* and *in vivo*. Therefore, the standard serological assays for measuring anti-sporozoite antibodies did not predict the enhanced levels of functional sporozoite neutralizing activity of serum from the mice immunized SS with AddaVax plus the combination of TLR agonists. The *in vitro* TSNA remained the best predictor of *in vivo* protection following challenge by the bite of infected mosquitoes.

## Discussion

Vaccines delivered by skin scarification may more closely mimic sporozoites delivered by the bite of infected mosquitoes, the gold standard for vaccines against the pre-erythrocytic stages of the Plasmodium parasite. The skin contains multiple populations of potential APC, including the Langerhans cells in the epidermis, multiple dendritic cell subpopulations in the dermis, along with innate immune cell populations such as NK cells and gamma delta T cells with important immunomodulatory functions. The innate and adaptive immune responses to sporozoites delivered into the skin have received intense scrutiny in recent years^39^,^40^,^41^. However, protozoan parasites as well as their arthropod vectors, have complex protein and antigenic repertoires that have evolved to modulate host immune responses^42^,^43^. Subunit peptide vaccines containing minimal T and B cell epitopes have the potential to circumvent these immune evasion strategies and more effectively focus the host immune response on known protective epitopes, such as the CS repeats which are the target of neutralizing antibodies^8^,^44^,^45^. Intravital imaging studies have demonstrated that antibodies against the CS repeats can provide a first line of host defense by immobilizing the sporozoites in the skin^9^ thus blocking parasite egress into the circulation and preventing the subsequent blood stage infection that is responsible for malaria morbidity and mortality. Therefore, investigation of potential induction and effector mechanisms of humoral immunity in the skin represent an important focus for vaccine development.

In previous murine studies, we demonstrated that topical application of Aldara, containing the TLR 7 agonist imiquimod, to the site of subcutaneously injected *P. falciparum* CS peptide, could elicit high levels of anti-repeat antibodies with sporozoite neutralizing activity that correlated with protection against challenge by mosquitoes infected with transgenic PfPb parasites expressing *P. falciparum* CS repeats^22^. Protection of the immunized mice was antibody mediated, as depletion of CD4+ T cells prior to challenge did not reduce resistance. In recent phase I/II studies of a seasonal flu vaccine, topical Aldara applied to the site of intradermally injected flu vaccine enhanced the seroconversion, magnitude and persistence of antibody and reduced flu-associated pathology^23^,^24^, supporting the potential utility of the murine model for assessing TLR agonist adjuvants despite the differences between rodent and human skin architecture and cellular components^46^.

In the current murine studies, we show that SS with CS peptide in Aldara can elicit high titers of anti-repeat antibodies in serum and CS-specific Th1- type CD4+ T cells in the spleen (Fig. 1A,C). SS with CS peptide in Aldara elicted a mixed Th1/Th2-type antibody response, consistent with the balanced IgG1 and IgG2c subtypes found in original studies using topical Aldara and subcutaneously injected CS peptide^22^. Importantly, SS with CS peptide in Aldara, but not CS peptide in PBS, elicited sporozoite neutralizing antibodies as measured by *in vitro* TSNA and reduced parasite burden in the liver following challenge by bites of PfPb infected mosquitoes (Figs 2A,B and 5A,B).

Efforts to increase adjuvant potency by addition of other TLR agonists to the Aldara crème formulation were not successful (Supplementary Fig. S1). A similar magnitude of anti-repeat antibody and sporozoite neutralizing activity was obtained following SS with CS peptide in Aldara with or without the TLR 3 agonist poly I:C. Moreover, although poly I:C has been shown to enhance immunogenicity of recombinant CS protein vaccines in mice and nonhuman primates^31^,^32^, SS with CS peptide and the TLR 3 agonist elicited only low levels of Th2-type IgG1 antibody and minimal Th1 CD4+ T cells in the spleen, similar to immune responses elicited by SS with CS peptide without adjuvant.

Modification of Aldara by addition of other TLR agonists was complicated by the presence of multiple excipients including emulsifiers and stabilizing agents, such as isostearic acid, polysorbate 60, sorbitan stearate, glycerol, methyl hydroxybenzoate, propyl hydroxybenzoate, xanthan gum and white soft paraffin, as well as a fixed high concentration of imiquimod^33^. As an alternative delivery vehicle, we investigated AddaVax, a squalene oil-in-water nano-emulsion similar in composition to the licensed MF59 adjuvant used in human vaccines^34^,^47^,^48^. However, SS with CS peptide in Addavax elicited a predominantly Th2-type IgG1 anti-repeat antibody response, in contrast to the balanced IgG1/IgG2c antibody responses elicited by SS with CS peptide in Aldara. More importantly, SS with CS peptide in AddaVax elicited minimal or no sporozoite neutralizating activity that was detectable *in vitro* or *in vivo* (Fig. 3). The addition of the TLR 7 agonist imiquimod, or the TLR 3 agonist poly I:C, to AddaVax did not alter the pattern of IgG1 skewed anti-repeat antibody, minimal IFNγ- secreting CD4+ T cells and lack of protection following challenge by bites of PfPb infected mosquitoes. Although imiquimod was a potent adjuvant in Aldara, the failure to enhance adjuvanticity when added to AddaVax was most likely related to the concentration of the TLR 7 agonist, with approximately a 10-fold higher concentration of imiquimod in Aldara as compared to the 125 μg added to AddaVax, the maximum recommended by the manufacturer. The high levels of imiquimod in Aldara have been associated with dermatitis and psoriasis-like lesions in a murine model^49^.

In contrast to imiquimod and poly I:C, the addition of the related imidazoquinoline, the TLR 7/8 agonist resiquimod, and/or the TLR 9 agonist CpG to AddaVax shifted the immune response to IgG2c antibody and a Th1-type cellular response (Fig. 4, Supplementary Fig. 2S). The Th1-type IgG subtype antibody elicited following addition of TLR 7/8 or TLR9 agonists to AddaVax was associated with increased levels of IFNγ- secreting CD4+ T cells in the spleen and a shift away from the IL5-producing Th2 cells elicited with AddaVax only. SS with peptide in AddaVax containing the TLR 7/8 and/or TLR9 agonists elicited levels of IgG2c anti-repeat antibodies comparable to those observed with Aldara (Table 1). However, despite the similar levels of antibody elicited by SS with AddaVax containing either a single or the combination of TLR agonists in ELISA assays, enhanced levels of sporozoite neutralizing antibody were measured by TSNA in serum of mice immunized SS with Addavax containing the combination of TLR agonists when compared to the single TLR agonists (Fig. 5A). The higher levels of sporozoite neutralizing antibodies measured by TSNA in serum of mice immunized SS with CS peptide in AddaVax containing a combination of TLR 7/8 and TLR 9 agonists correlated with greater reduction of liver stage parasites following challenge (Fig. 5B).

It remains to be determined whether enhanced neutralizing antibodies obtained by SS using a combination of TLR agonists reflects an additive or synergistic effect. Synergy between multiple TLR agonists has been shown to be a critical factor in the potent immunogenicity of attenuated viral vaccines as well subunit vaccines^50^,^51^. Engagement of multiple TLR by parasite PAMPs also play a role in the induction of natural immunity to malaria blood stages in endemic areas^42^. Although TLR distribution varies among mammalian species^52^,^53^, it is encouraging that a similar TLR 7/8 and TLR 9 agonist combination was a potent adjuvant for enhancing antibody titers to a recombinant HIV vaccine in nonhuman primates^35^.

The efficacy of SS for vaccine delivery most likely reflects not only PAMPS provided by TLR agonists but also damage associated molecular patterns (DAMP) signals provided by scarification. In previous studies, we found that topical application of peptide in Aldara, without skin scarification, was poorly immunogenic with GMT 640 following four applications (R. Mitchell, unpublished). SS has been shown in other vaccine models to elicit cellular immune responses not found following subcutaneous or intradermal immunization^25^. In the current studies, SS with CS peptide without adjuvant elicited antibody, presumably due to DAMPs signals resulting from tissue damage by SS that initiated antibody responses^54^,^55^. However, SS with CS peptide without adjuvant elicited low titers of predominantly Th2-type IgG1 antibody that did not neutralize sporozoites *in vitro* nor protect against challenge *in vivo* (Figs 1, 2, 4 and 5). Similarly, SS with CS peptide in AddaVax, while significantly increasing (>4 fold) the magnitude of the antibody titer, also elicited a predominantly Th2-type IgG1 antibody response with minimal or no neutralizing antibody or protection against challenge (Figs 3, 4 and 5).

The mechanisms whereby the addition of TLR 7/8 and 9 agonists to AddaVax and the resultant shift to Th1-type IgG2c antibody provide enhanced sporozoite neutralizing antibody and protection most likely reflects enhancement of both quantity and quality of the anti-repeat antibody response. IgG subtypes have distinct functional roles *in vitro* and *in vivo* that could contribute to vaccine efficacy. Early murine studies demonstrated that IgG2c opsonizing antibodies can enhance phagocytosis and killing of sporozoites^56^,^57^. Nevertheless, in the current studies, sporozoite neutralizing activity measured *in vitro* in the absence of phagocytic cells in TSNA correlated with resistance to sporozoite challenge *in vivo* (Fig. 5). Moreover, anti-repeat MAB of all IgG subtypes are protective^58^ and passive transfer of Fab fragments of repeat specific MAB can protect mice against sporozoite challenge^7^, suggesting that IgG FcR mediated interactions are not critical for function of sporozoite neutralizing antibodies.

While addition of the TLR agonists increased antibody responses, the overall magnitude of the response as reflected in ELISA GMT did not correlate with the increased neutralizing capacity of antibodies elicited by SS with AddaVax containing the combination of TLR agonists (Table 1). The total amounts of anti-repeat antibody elicited by SS with peptide in AddaVax plus CpG (101 μg/ml) or resiquimod (250 μg/ml) was similar to that elicited by the combination of resiquimod +CpG (256 μg/ml). Nevertheless, SS with the combination of TLR agonists, but not the single TLR agonists, elicited higher levels of sporozoite neutralizing antibody detected *in vitro* and lower parasite burden *in vivo* following challenge (Fig. 5). In addition to increasing the magnitude of the antibody response, the addition of TLR agonists to adjuvants has been shown to increase the breadth and affinity of the antibody to malaria blood stage antigens^37^. However, the magnitude and fine specificity of anti-repeat antibodies elicited by SS with CS peptide in AddaVax+ resiquimod were similar to those obtained with AddaVax plus the combination of TLR agonists (Table 1). Moreover, the affinity of antibodies elicited by SS with the combination of TLR agonists was similar to single TLR agonists when measured by chaotropic elution assays, the ability to cross-link CS on viable PfPb sporozoites as measured by the CSP reaction, or IC50 in a MAB 2A10 competition assay (Table 1, Supplementary Fig. S5). Therefore, the standard serological assays for measuring anti-sporozoite antibodies did not correlate with neutralizing antibody activity and the TSNA remains the best predictor of protection *in vivo*. The cellular origin of polarizing cytokines/chemokines and the interaction of T:B cells in skin draining lymph nodes are under investigation to explore potential cellular factors that contribute to the induction of enhanced levels of neutralizing antibodies by combinations of TLR agonists.

The use of well-defined TLR agonists as adjuvants can also advance the rational design of adjuvants for specific vaccine target populations, such as the pediatric population that suffers the majority of the morbidity and mortality due to *P. falciparum* infection. In the recent Phase III clinical trials of the RTS,S malaria vaccine in Africa, lower immunogenicity was observed in young infants (6–12 weeks) when compared to older infants (5–17 months)^59^. While numerous factors may have contributed to the reduced immunogenicity of RTS,S vaccine in neonates, TLR distribution on neonatal cells is known to differ from that in older children and adults^60^. The finding that the TLR 7/8 agonist resiquimod is functional in neonatal cells^61^ raises the hope that rational design of TLR agonist-based adjuvants can be used to enhance the immunogenicity of pediatric malaria vaccines.

In addition to adjuvant, the vaccine dose plays a critical role in immunogenicity. Preliminary experiments using a sensitive 2-site assay to measure antigen in skin extracts obtained from SS sites, suggest that <10% of the 50 μg peptide dose is delivered into the skin by scarification. Peptide concentration in the skin can potentially be increased by the use of microneedles and patches^62^,^63^ which can contain up to 100 microneedles/array, to increase immunogenicity. Nevertheless, the simple two pronged stylet provides an inexpensive facile method for delivery of vaccines to the skin to allow rapid screening of peptide immunogens and adjuvant formulations for use in next generation skin delivery systems.

The current studies demonstrate that SS with CS peptide in combination with TLR agonists can induce systemic neutralizing antibody with the potential to block parasite egress from the skin and invasion of liver cells, thus preventing initiation of the Plasmodium erythrocytic cycle responsible for malaria morbidity and mortality. These studies support efforts to optimize SS delivered malaria vaccines to elicit Th1-type antibodies to target not only sporozoites but also to enhance opsonization and phagocytosis of blood stage parasites responsible for clinical disease^64^,^65^. SS thus has the potential to provide an easily delivered malaria vaccine to elicit antibodies that target the parasite at multiple stages in its complex life cycle.

## Methods

### Immunization

C57Bl/6 female mice (Jackson Labs) 6–8 weeks of age were immunized by skin scarification (SS) at 14–21 day intervals with four to five doses of a synthetic peptide representing the repeats of the *P. falciparum* circumsporozoite (CS) protein^27^,^66^. The CS repeats contain a well defined protective B cell epitope and H-2^b^ restricted CD4+ T cell epitope, but no class I restricted CD8+ T cell epitopes. The CS peptide (50 μg) in various adjuvant formulations (total volume of 100–200 μl) was applied to a 2 cm^2^ area of unshaved scapular dorsal skin by skin scarification using 10 pricks with a two-pronged stylet (Precision Medical Inc., Denver, PA) as used in smallpox vaccination. Mice were bled prior to each immunization and 14 days after the final boost. Serum was stored at −20 °C until used in serological assays. The study was conducted in strict accordance with the recommendations in the Guide for the Care and Use of Laboratory Animals of the National Institutes of Health. The protocol was approved by the Institutional Animal Care and Use Committee, NYU School of Medicine (Protocol number 1501081).

### Adjuvants

The TLR agonists tested as adjuvants focused on the endosomal TLRs which include TLR 7, 8 and 9, which signal through MyD88, and TLR 3 which signals through the alternate TRIF pathway^53^,^66^,^67^,^68^. The agonists were added either to the Aldara cream formulation containing imiquimod (3 M, St. Paul, MN) or to AddaVax an oil-in-water adjuvant (InvivoGen). Aldara is FDA approved for topical treatment of human dermatologic skin conditions such as genital warts, actinic keratosis and superficial basal cell carcinomas. One tenth of each Aldara packet of 5% cream containing 12.5 mg of imiquimod was mixed with CS peptide and applied to the skin prior to SS. AddaVax (InvivoGen), is a squalene oil-in water nano-emulsion that is comparable to MF59 adjuvant licensed by FDA for use in a seasonal flu vaccine^69^,^70^. Synthetic TLR agonists tested to enhance adjuvanticity included the imidazoquinolines imiquimod and resiquimod, which are TLR 7 and TLR 7/8 agonists, respectively, the TLR 3 agonist polyriboinosinic polyribocytidylic acid (poly I:C) that mimics double stranded viral RNA (all InvivoGen Vaccigrade) and the TLR 9 agonist CpG ODN a cytosine: guanine oligodeoxyribonucleotide that mimics an unmethylated bacterial DNA motif (The Certified Midland Reagent Co., MidlandTX). The TLR agonists were used at the manufacturer’s recommended maximum dose per mouse (range 125–150 μg).

### Serological Assays

IgG anti-repeat antibody titer was measured by ELISA using two-fold dilutions of individual serum collected 14 days post each immunization and plates coated with the (T1B)_4_ peptide immunogen, which contains both the minor and major repeats found in the *P. falciparum* CS protein^27^. Fine specificity was determined using plates coated with peptides representing either the CS minor 5’repeat (DPNANPNV)_2_ or major repeat (NANP)_3_. Results are expressed as geometric mean titers (GMT) with the endpoint defined as the final dilution giving an OD greater than three times the OD of BSA-coated control wells. Total anti-repeat antibody concentration (μg/ml) was calculated by ELISA using pooled sera tested at two-fold dilutions and known concentrations of monoclonal anti-repeat MAB 2A10 antibody as standard^71^. The ability of immune serum to compete with biotinylated -MAB 2A10 for binding to CS repeats was tested in a competition ELISA using two-fold dilutions of pooled serum (starting concentration 50 ug/ml) to determine the IC50. IgG subtypes were determined in a repeat peptide ELISA using MAB specific for murine Th2-associated IgG1 antibody or Th1- associated IgG2c antibody (Southern Biotechnology, AL). Affinity of anti-repeat antibody was determined using pooled serum adjusted to give an OD+ 1.5 and determining concentration of chaotropic NH_4_SCN required to give 50% reduction in OD. Reactivity with viable PfPb sporozoites was determined by Circumsporozoite Precipitin (CSP) assay with endpoint titer defined as the last serum dilution giving positive precipitin reaction on 10% of sporozoites, as determined by phase microscopy^38^.

### Cellular Assays

CS-specific T cells were measured in pooled spleens (3–5 mice/group) obtained following the final SS immunization. Splenocytes were stimulated with immunogen, medium only or PMA/Ionomycin as positive control (Sigma, St. Louis, MO). ELISPOT kits (BD Biosciences, CA) specific for murine IFNγ and IL5 were used to assess Th1- and Th2-type responses, respectively. Results are expressed as spot forming cells (SFC)/10^6^ spleen cells after subtraction of media control background. The phenotype of the cytokine producing T cells was determined using anti-CD4 (MAB GK 1.5) or anti-CD8 (MAB 2.43) monoclonal antibodies (Bio X Cell, Lebanon, NH).

### Sporozoite Neutralizing Antibody

Functional antibody reactive with viable sporozoites was measured *in vitro* using the Transgenic Sporozoite Neutralization Assay (TSNA) based on PfPb, a transgenic *P. berghei* parasite in which the *P. berghei* CS repeat region has been replaced with *P. falciparum* CS repeats^45^,^72^. PfPb sporozoites (2 × 10^4^) were incubated for 40 min on ice with or without murine serum (1:5–1:40 dilution) prior to addition to confluent cultures of human HepG2 hepatoma cells. Controls included PfPb sporozoites incubated with 25 μg/ml of MAB 2A10 specific for *P. falciparum* CS repeats or MAB 3D11 specific for *P. berghei* CS repeats as positive and negative controls, respectively. After 48 hours of culture, the HepG2 cells were harvested, total RNA was extracted using a PureLink RNA Mini Kit (Life technologies, Grand Island, NY), reverse transcribed to cDNA and parasite levels determined by realtime qPCR using primers specific for *P. berghei* 18 S rRNA and SyBr Green (Qiagen)^22^,^73^. Results are expressed as number of 18S rRNA copies based on an 18S rRNA plasmid reference standard.

### Challenge

SS immunized mice and naïve controls were challenged by placing individual anesthetized mice ventral side down on a small cage of PfPb infected mosquitoes to deliver 10–15 infected bites. Livers were dissected at 40 h. post challenge and homogenized in TRI Reagent (Molecular Research Center, Inc., Cincinnati, OH) with a tissue homogenizer (Fisher Scientific, Pittsburgh, PA). Total RNA was extracted and reverse transcribed to cDNA and 18S rRNA parasite copy numbers were determined by realtime qPCR, as described above^22^. As previous studies using injection of known numbers of sporozoites demonstrated that >90% reduction of liver stage burden was associated with sterile immunity or delayed prepatent period, reduction of parasite levels >90% when compared to naïve mice was considered biologically significant^1^,^30^.

### Statistics

Statistical analysis was carried out using GraphPad Prism software version 6.07. Differences between experimental groups versus naïve controls were determined by Kruskal-Wallis one-way analysis of variance with Dunn’s multiple comparison test. For analysis of parasite 18S rRNA copy number measured in TSNA and following challenge, the average copy number for each vaccination group was calculated across each trial (performed in triplicate) within each group. Correlations of functional immunity were based on > 90% reduction in rRNA copy number evaluated by Pearson Correlation Coefficient. A P value < 0.05 was considered significant. A > 4-fold difference in antibody geometric mean titers was considered significant.

## Additional Information

**How to cite this article**: Mitchell, R. A. *et al.* Skin scarification with *Plasmodium falciparum* peptide vaccine using synthetic TLR agonists as adjuvants elicits malaria sporozoite neutralizing immunity. *Sci. Rep.* **6**, 32575; doi: 10.1038/srep32575 (2016).

## Supplementary Material

Supplementary Information

## Figures and Tables

**Table 1:** Fine Specificity of antibody elicited by SS with CS peptide in Aldara or Addavax with or without TLR agonists.

SS Adjuvant Composition		Anti-Repeat Antibody						
Delivery vehicle	TLR agonist	GMT a (range)	ug/ml^b^	Fine Specificity^c^		IgG2c/IgG1 Ratio^d^	Affinity^e^ [M]	CSP^f^ Titer
				Minor	Major			
Aldara	TLR 7 imiquimod	142,631 (81,920–327,640)	413	163,840	40,960	0.967	0.625	80
Addavax	none	20,480 (5,120–40,960)	55	20,480	5,120	0.022	0.625	40
Addavax	TLR 7/8 resiquimod	124,168 (81,920–327,640)	250	81,920	40,960	1.117	0.938	40
Addavax	TLR 9 CpG	47,044 (20,480–81,920)	101	40,960	5,120	0.698	0.469	40
Addavax	TLR 7/8 resiquimod + TLR 9 CpG	142,631 (81,920–327,640)	256	81,920	40,960	1.217	0.469	80

^a^ELISA geometric mean titer (GMT) and range of endpoints in individual serum (5 mice/group) obtained post fourth SS immunization.

^b^Total μg/ml of anti-repeat antibody in pooled serum calculated based on MAB 2A10 standard.

^c^ELISA plates coated with peptide representing the CS minor 5’repeat region (DPNANPNV)_2_ or CS major repeat region (NANP)_3_.

^d^Ratio of IgG2c/IgG1 anti-repeat antibody OD in pooled serum (1:2560 dilution).

^e^Antibody affinity as measured by molar concentration of NH_4_SCN required to elute 50% of anti-repeat antibody in ELISA.

^f^Endpoint dilution of pooled serum giving positive Circumsporozoite Precipitin (CSP) reaction with viable PfPb sporozoites.
