# Supplementary material for: Skin scarification with Plasmodium falciparum peptide vaccine using synthetic TLR agonists as adjuvants elicits malaria sporozoite neutralizing immunity
Source: Sci Rep. 2016 Sep 14;6:32575. doi: 10.1038/srep32575 (PMC5021941; doi:10.1038/srep32575)
Supplement: Supplementary Information [file srep32575-s1.pdf]

Supplementary Figures:

Skin scarification with *Plasmodium falciparum* peptide vaccine using synthetic TLR agonists as adjuvants elicits malaria sporozoite neutralizing immunity

Robert A. Mitchell, Rita Altszuler, Ute Frevert and Elizabeth H. Nardin*
Department of Microbiology, Division of Parasitology, New York University School of Medicine, New York, NY, USA

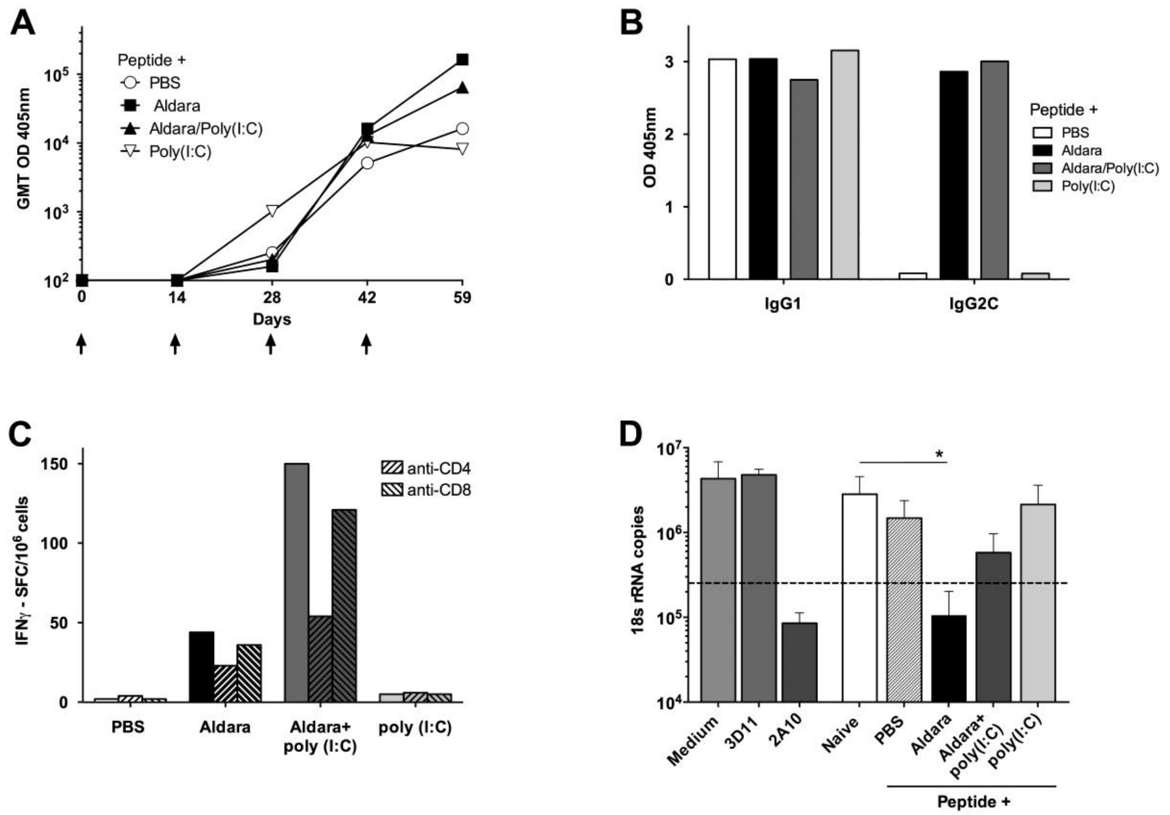

Supplementary Figure S1. Combination of Aldara containing the TLR 7 agonist imiquimod and TLR 3 agonist poly I:C does not increase immunogenicity of SS administered vaccine. (A) IgG anti-repeat antibody GMT measured by ELISA using serum (5 mice/group) obtained following each SS immunization (arrows) with CS peptide in PBS, in Aldara containing the TLR7 agonist imiquimod, in Aldara plus poly I:C, or with CS peptide mixed with poly I:C only. (B) IgG subtypes of anti-repeat antibodies in pooled sera (1:320 dilution) obtained following four SS immunizations. (C) IFNγ ELISPOT (SFC / 10^6 spleen cells) measured in the absence (solid bars) or presence of anti-CD4 or anti-CD8 MAB (hatched bars). Minimial SFCs were found in Adjuvant only groups: 3 SFCs in Aldara only group, 10 SFCs in poly I:C only group, and 7 SFCs in Aldara + poly I:C only group. (D) TSNA carried out using PfPb sporozoites incubated with 1:5 dilution of serum from individual mice following four SS immunizations with CS peptide in PBS, Aldara , Aldara plus

poly I:C, or poly I:C only. Results shown as mean parasite 18s rRNA in HepG2 cell culture extracts obtained at 48 h post addition of PfPb sporozoites. Dotted line indicates 90% reduction of parasite 18S rRNA copy numbers. *$P<0.05$; Kruskal-Wallis test with Dunn's multiple comparison between groups against naïve. Error bars are SD for all qPCR replicates.

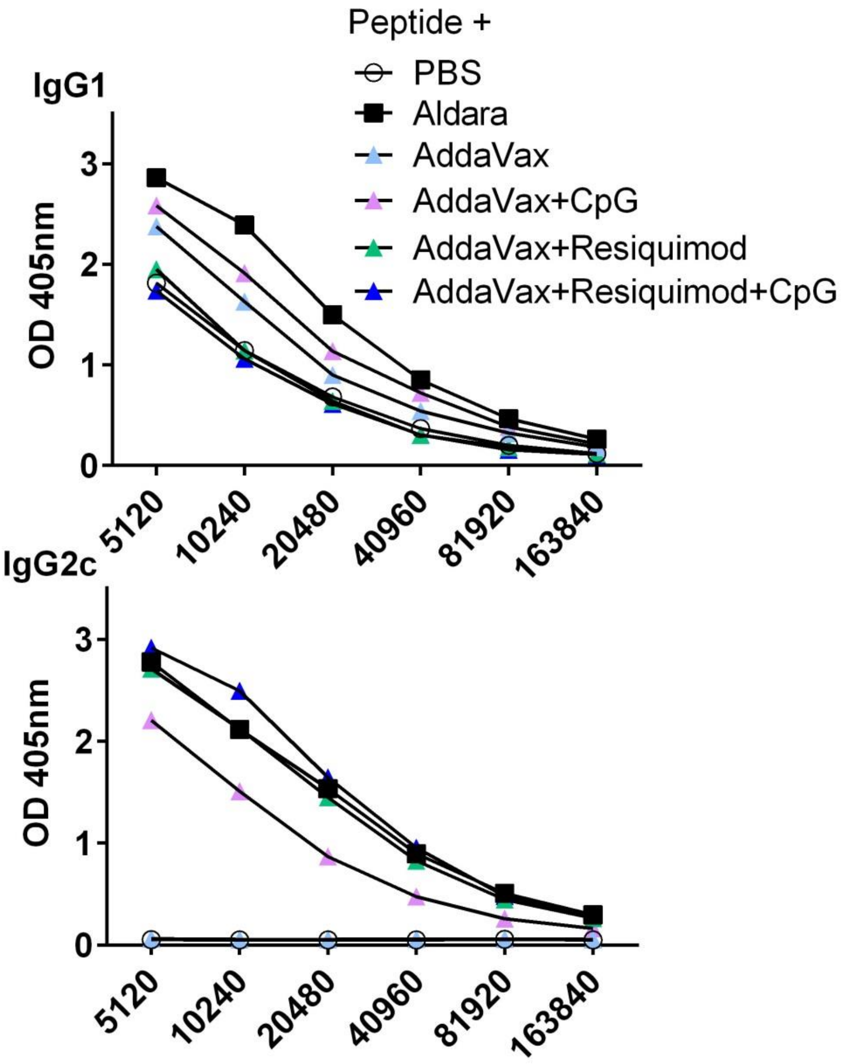

Supplementary Figure S2: Anti-repeat antibody IgG subtypes. Two fold dilutions of pooled sera obtained after four SS immunizations was titrated (starting at a 5120 dilution) against repeat peptide followed by enzyme labeled MAB specific for murine IgG1 and IgG2c.

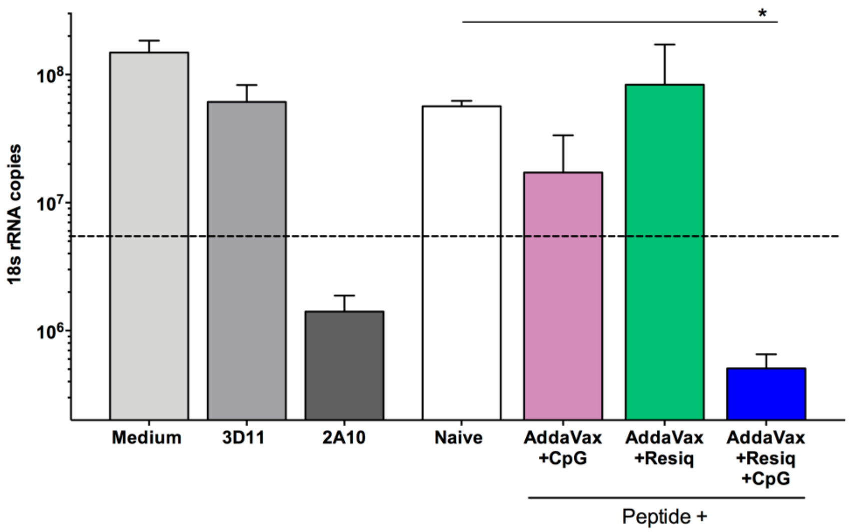

Supplementary Figure S3. SS with CS peptide in AddaVax containing a combination of TLR 7/8 and TLR 9 agonists elicits enhanced sporozoite neutralizing antibodies. TSNA was carried out using 1:5 dilution of individual serum (3 mice/group) following four SS immunizations with CS peptide in Addavax containing either the TLR 9 agonist CpG or the TLR 7/8 agonists resiquimod or a combination of both CpG and resiquimod. Error bars are SD of all qPCR replicates for assay controls and immune groups. Differences in 18S rRNA copy number in experimental versus naïve groups analyzed by Kruskal-Wallis with Dunn's multiple comparison test *$P<0.05$; Kruskal-Wallis test with Dunn's multiple comparison between groups against naïve. Error bars are SD for all qPCR replicates.

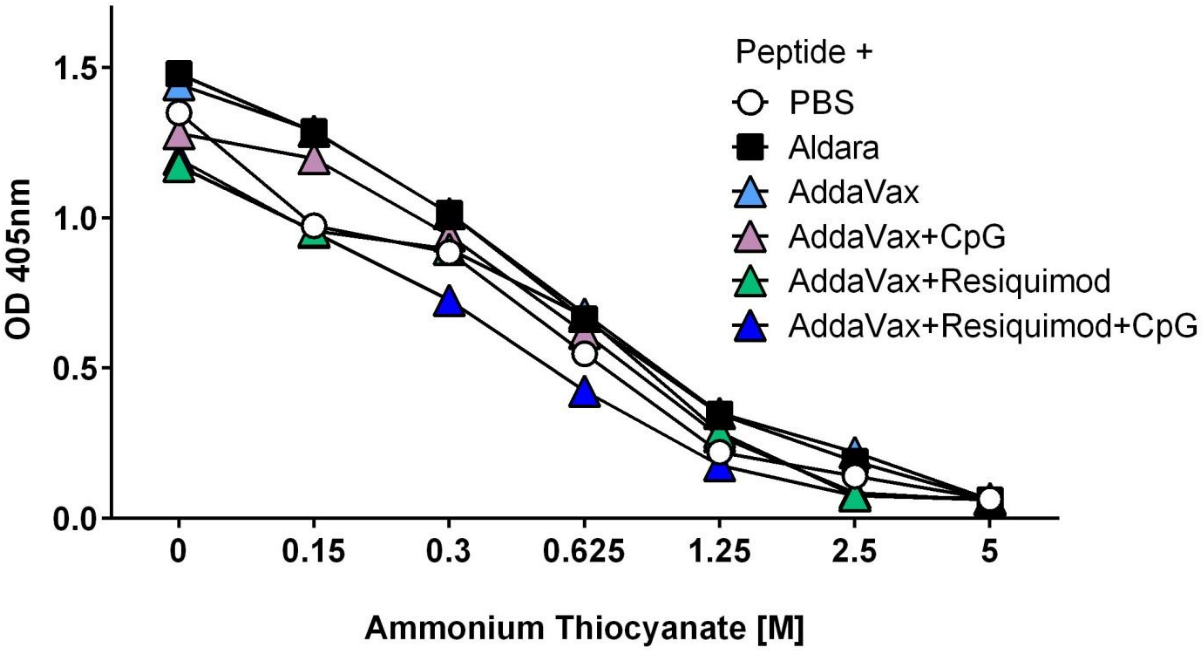

Supplementary Figure S4: Affinity of anti-repeat antibodies elicited by SS immunization. Pooled sera from each experimental group were adjusted to give an OD $\pm$ 1.5 in repeat peptide ELISA (dilution 1:1280 – 1:20480). Following incubation of serum with repeat peptide-coated wells, two-fold dilutions of chaotropic NH_4SCN were added to plates. Affinity was determined by calculating the molar concentration of NH_4SCN required for 50% reduction of OD (Table 1).

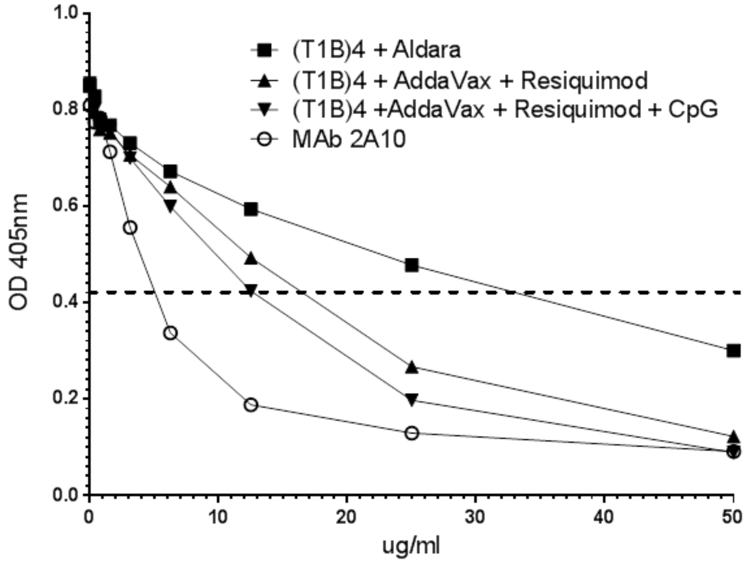

IC50:

(T1B)4 + Aldara	= 34.0 ug/ml
(T1B)4 + AddaVax + Resiquimod	= 16.7 ug/ml
(T1B)4 + AddaVax + Resiq + Cpg	= 14.4 ug/ml
Monoclonal Ab 2A10	= 5.3 ug/ml

Supplementary Figure S5: IC50 of Immune Serum Determined in Competition Assay.

Two fold dilutions of pooled immune sera (starting concentration 50 µg/ml) (closed symbols) or MAB 2A10 (open symbol) were tested in repeat peptide ELISA to determine amount of antibody required to inhibit 50% of binding of biotinylated MAB 2A10 (0.0156 µg/ml).
